# Supplementary material for: Phosphopeptide binding by Sld3 links Dbf4‐dependent kinase to MCM replicative helicase activation
Source: EMBO J. 2016 Feb 24;35(9):961–73. doi: 10.15252/embj.201593552 (PMC4864760; doi:10.15252/embj.201593552)
Supplement: Supplementary file 1 — Appendix [file EMBJ-35-961-s001.pdf]

Appendix for

Phosphopeptide binding by Sld3 links Dbf4-dependent kinase to MCM replicative helicase activation

Tom D. Deegan, Joseph T.P. Yeeles and John F. X. Diffley

correspondence to: john.diffley@crick.ac.uk

This appendix includes:

Supplementary Text
Tables S1 to S2

Supplementary Text | Identification of Mcm 4 and 6 phosphorylation sites required for Sld3/7 binding

To isolate the Sld3 interacting region in Mcm6, we purified a version of MCM in which Mcm6 could be separated into its N and C-terminal domains by cleavage with TEV protease (Fig. EV4A). Supplementary Fig. EV4B shows that Sld3 interacts with the N-terminal portion of Mcm6.

The majority of phosphorylation sites identified to date within Mcm6 are located in the extreme N-terminus of the protein (Fig. EV4A). We next constructed mutants in which the first 84 amino acids of Mcm6 were deleted (ΔN), or in which 25 potential DDK phosphorylation sites in this region were mutated to alanine (25A) (Randell et al., 2010). Both of these mutants showed a partial defect in Sld3 binding (Fig. EV4C). To identify additional phosphorylation sites in Mcm6 responsible for the residual binding of Mcm6ΔN to Sld3, we tested for the binding of Sld3 to arrays of 18-residue phosphorylated peptides covering the region of Mcm6 shown to interact with Sld3. 18 potential Sld3-interacting serine and threonine phosphorylation sites were identified, as is summarised in Fig. EV5B. Mutation of 11 of these sites to alanine, in combination with deletion of residues 2-84, produced an Mcm6 mutant (ΔN+11A) that was incapable of binding Sld3 (Fig. 4F). A number of Mcm6 mutants containing different subsets of these sites mutated to alanine showed only partial defects in Sld3 binding (Fig. EV5C-F).

To isolate an Sld3-binding mutant in Mcm4, we used a previously described phosphorylation site mutant of Mcm4, Mcm4 25A, that had been reported to exhibit a slow growth phenotype (Randell et al., 2010). A number of the serines and threonines mutated in this allele interacted with Sld3 on a peptide array (Fig. EV5G,H).

To generate phospho-mimicking mutants in Mcm4/6, the serine and threonine residues mutated to alanine in the respective 25A mutants were replaced with aspartate residues.

Table S1 | *S. cerevisiae* strains

Strain Name	Genotype	Reference
yMD7	*MAT**a** ade2-1 ura3-1 his3-11,15 trp1-1 leu2-3,112 can1-100 bar1::Hyg pep4::KanMX*<br>*MCM6::MCM6-3xFLAG (Nat-NT2)*	Generated in the laboratory
yTD13	*MAT**a** ade2-1 ura3-1 his3-11,15 trp1-1 leu2-3,112 can1-100 bar1::Hyg pep4::KanMX*<br>*MCM6::MCM6-3xFLAG (Nat-NT2)*<br>*his3::HIS3pRS303/CDT1,GAL4*<br>*trp1::TRP1pRS304/MCM4, MCM5 leu2::LEU2pRS305/MCM6Δ2-84, MCM7*<br>*ura3::URA3pRS306/MCM2, CBP-MCM3*	This study
yTD24	*MAT**a** ade2-1 ura3-1 his3-11,15 trp1-1 leu2-3,112 can1-100 bar1::Hyg pep4::KanMX*<br>*MCM6::MCM6-3xFLAG (Nat-NT2)*<br>*his3::HIS3pRS303/CDT1,GAL4*<br>*trp1::TRP1pRS304/MCM4, MCM5 leu2::LEU2pRS305/MCM6TEV2, MCM7*<br>*ura3::URA3pRS306/MCM2, CBP-MCM3*	This study
yTD29	*MAT**a** ade2-1 ura3-1 his3-11,15 trp1-1 leu2-3,112 can1-100 bar1::Hyg pep4::KanMX*<br>*MCM6::MCM6-3xFLAG (Nat-NT2)*<br>*his3::HIS3pRS303/CDT1,GAL4*<br>*trp1::TRP1pRS304/MCM4, MCM5 leu2::LEU2pRS305/MCM6 12A, MCM7*<br>*ura3::URA3pRS306/MCM2, CBP-MCM3*	This study
yTD30	*MAT**a** ade2-1 ura3-1 his3-11,15 trp1-1 leu2-3,112 can1-100 bar1::Hyg pep4::KanMX*<br>*MCM6::MCM6-3xFLAG (Nat-NT2)*<br>*his3::HIS3pRS303/CDT1,GAL4*<br>*trp1::TRP1pRS304/MCM4, MCM5 leu2::LEU2pRS305/MCM6Δ2-84+11A, MCM7*<br>*ura3::URA3pRS306/MCM2, CBP-MCM3*	This study
yTD33	*MAT**a** ade2-1 ura3-1 his3-11,15 trp1-1 leu2-3,112 can1-100 bar1::Hyg pep4::KanMX*<br>*MCM6::MCM6-3xFLAG (Nat-NT2)*<br>*his3::HIS3pRS303/CDT1,GAL4*<br>*trp1::TRP1pRS304/MCM4, MCM5 leu2::LEU2pRS305/MCM6Δ2-84+Δ199-260, MCM7 ura3::URA3pRS306/MCM2, CBP-MCM3*	This study
yTD39	*MAT**a** ade2-1 ura3-1 his3-11,15 trp1-1 leu2-3,112 can1-100 bar1::Hyg pep4::KanMX*<br>*MCM6::MCM6-3xFLAG (Nat-NT2)*<br>*his3::HIS3pRS303/CDT1,GAL4*<br>*trp1::TRP1pRS304/MCM4, MCM5 leu2::LEU2pRS305/MCM6Δ2-84+3A, MCM7*<br>*ura3::URA3pRS306/MCM2, CBP-MCM3*	This study
yTD49	*MAT**a** ade2-1 ura3-1 his3-11,15 trp1-1 leu2-3,112 can1-100 bar1::Hyg pep4::KanMX*<br>*MCM6::MCM6-3xFLAG (Nat-NT2)*<br>*his3::HIS3pRS303/CDT1,GAL4*<br>*trp1::TRP1pRS304/MCM4, MCM5 leu2::LEU2pRS305/MCM6 25A, MCM7*<br>*ura3::URA3pRS306/MCM2, CBP-MCM3*	This study
yTD54	*MAT**a** ade2-1 ura3-1 his3-11,15 trp1-1 leu2-3,112 can1-100 bar1::Hyg pep4::KanMX*<br>*MCM4::MCM4-3xFLAG (Nat-NT2)*<br>*his3::HIS3pRS303/CDT1,GAL4*<br>*trp1::TRP1pRS304/MCM4 25A, MCM5 leu2::LEU2pRS305/MCM6, MCM7*<br>*ura3::URA3pRS306/MCM2, CBP-MCM3*	This study

yTD60	*MAT**a** ade2-1 ura3-1 his3-11,15 trp1-1 leu2-3,112 can1-100 bar1::Hyg pep4::KanMX*<br>*MCM4::MCM4-3xFLAG (ADE2)*<br>*MCM6::MCM6-3xFLAG (Nat-NT2)*<br>*his3::HIS3pRS303/CDT1,GAL4*<br>*trp1::TRP1pRS304/MCM4 25A, MCM5 leu2::LEU2pRS305/MCM6Δ2-84+11A, MCM7 ura3::URA3pRS306/MCM2, CBP-MCM3*	This study
yTD61	*MAT**a** ade2-1 ura3-1 his3-11,15 trp1-1 leu2-3,112 can1-100 bar1::Hyg pep4::KanMX*<br>*MCM6::MCM6-3xFLAG (Nat-NT2)*<br>*his3::HIS3pRS303/CDT1,GAL4*<br>*trp1::TRP1pRS304/MCM4 14D, MCM5 leu2::LEU2pRS305/MCM6 25D, MCM7 ura3::URA3pRS306/MCM2, CBP-MCM3*	This study
yTD62	*MAT**a** ade2-1 ura3-1 his3-11,15 trp1-1 leu2-3,112 can1-100 bar1::Hyg pep4::KanMX*<br>*MCM6::MCM6-3xFLAG (Nat-NT2)*<br>*his3::HIS3pRS303/CDT1,GAL4*<br>*trp1::TRP1pRS304/MCM4, MCM5 leu2::LEU2pRS305/MCM6 25D, MCM7 ura3::URA3pRS306/MCM2, CBP-MCM3*	This study
yTD65	*MAT**a/**α ade2-1/ ade2-1 ura3-1/ ura3-1 his3-11,15/ his3-11,15 trp1-1/ trp1-1 leu2-3,112/ leu2-3,112 can1-100/ can1-100 $SLD3^{+}$/sld3-6E*	This study
yTD69	*MAT**a** ade2-1 ura3-1 his3-11,15 trp1-1 leu2-3,112 can1-100 bar1::Hyg pep4::KanMX*<br>*MCM4::MCM4-3xFLAG (Nat-NT2)*<br>*his3::HIS3pRS303/CDT1,GAL4*<br>*trp1::TRP1pRS304/MCM4 25D, MCM5 leu2::LEU2pRS305/MCM6, MCM7 ura3::URA3pRS306/MCM2, CBP-MCM3*	This study
yTD70	*MAT**a** ade2-1 ura3-1 his3-11,15 trp1-1 leu2-3,112 can1-100 bar1::Hyg pep4::KanMX*<br>*MCM4::MCM4-3xFLAG (ADE2)*<br>*MCM6::MCM6-3xFLAG (Nat-NT2)*<br>*his3::HIS3pRS303/CDT1,GAL4*<br>*trp1::TRP1pRS304/MCM4 25D, MCM5 leu2::LEU2pRS305/MCM6 25D, MCM7 ura3::URA3pRS306/MCM2, CBP-MCM3*	This study
yTD71	*MAT**a** ade2-1 ura3-1 his3-11,15 trp1-1 leu2-3,112 can1-100 cdc7-4*<br>*MCM4::mcm4-25D (KanMx)*	This study
yTD72	*MAT**a** ade2-1 ura3-1 his3-11,15 trp1-1 leu2-3,112 can1-100*<br>*MCM4::mcm4-25A (KanMx)*	This study
yTD73	*MATα ade2-1 ura3-1 his3-11,15 trp1-1 leu2-3,112 can1-100*<br>*MCM6::mcm6Δ2-84+11A (Nat-NT2)*	This study
yTD74	*MAT**a/**α ade2-1/ ade2-1 ura3-1/ ura3-1 his3-11,15/ his3-11,15 trp1-1/ trp1-1 leu2-3,112/ leu2-3,112 can1-100/ can1-100 mcm4-25A (KanMx)/$MCM4^{+}$ $MCM6^{+}$/mcm6Δ2-84+11A (Nat-NT2)*	This study
yTD75	*MATα ade2-1 ura3-1 his3-11,15 trp1-1 leu2-3,112 can1-100 cdc7-4*<br>*MCM6::mcm6-18D (KanMX)*	This study
yTD76	*MAT**a/**α ade2-1/ ade2-1 ura3-1/ ura3-1 his3-11,15/ his3-11,15 trp1-1/ trp1-1 leu2-3,112/ leu2-3,112 can1-100/ can1-100 $SLD3^{+}$/sld3-2E3*	This study

Table S2 | Plasmids generated in this study

Name	Cloning vector*	Insert	Generation of insert	5′ site	3′ site
pTD12	pJF4	*MCM6Δ2-84*	PCR (*S. cerevisiae* W303 genomic DNA template)	SgrA1	NotI
pTDP1	pJF4	*MCM6-T150A*	Synthetic construct (*MCM6-1-209)*	SgrA1	PshA1
pTD24	pJF4	*MCM6-TEV2*	Synthetic construct (*MCM6-209-588)*	PshA1	SnaB1
pTD29	pTDP1	*MCM6-11A*	Synthetic construct (*MCM6-209-588)*	PshA1	SnaB1
pTD30	pTD12	*MCM6-11A*	Synthetic construct (*MCM6-209-588)*	PshA1	SnaB1
pTD33	pJF4	*MCM6Δ2-84, Δ199-260*	Synthetic construct (*MCM6-1-588)*	SgrA1	SnaB1
pTD39	pTD12	*MCM6-3A*	Synthetic construct (*MCM6-209-588)*	PshA1	SnaB1
pTD49	pJF4	*MCM6-25A*	Synthetic construct (*MCM6-1-209)*	SgrA1	PshA1
pTD54	pJF3	*MCM4-25A*	Synthetic construct (*MCM4-1-450)*	SgrA1	MluI
pTD58	pJF3	*MCM4-14D*	Synthetic construct (*MCM4-1-450)*	SgrA1	MluI
pTD62	pJF4	*MCM6-25D*	Synthetic construct (*MCM6-1-209)*	SgrA1	PshA1
pTD69	pJF3	*MCM4-25D*	Synthetic construct (*MCM4-1-450)*	SgrA1	MluI
pTD71a	pJF3	*MCM4-25D (+500bp upstream)*	Synthetic construct (*MCM4-1-450)*	SgrA1	MluI
pTD71b	pFA6a-kanMX6	*MCM4-25D (+500bp upstream)*	PCR (pTD71a template)	NdeI	PacI
pTD72a	pJF3	*MCM4-25A (+500bp upstream)*	Synthetic construct (*MCM4-1-450)*	SgrA1	MluI
pTD72b	pFA6a-kanMX6	*MCM4-25A (+500bp upstream)*	PCR (pTD72a template)	NdeI	PacI
pTD73a	pFA6a-natNT2	*MCM6 (+500bp upstream)*	PCR (*S. cerevisiae* W303 genomic DNA template)	NdeI	PacI
pTD73b	pTD73a	*MCM6Δ2-84+11A*	PCR of *MCM6-1-587* (pTD30 template)	AfiII	SnaB1
pTD75	pTD73a	*MCM6-25D*	PCR of *MCM6-1-587* (pTD62 template)	AfiII	SnaB1
pGEX-6p-1/*SLD7*	pGEX-6p-1	*SLD7*	PCR (*S. cerevisiae* W303 genomic DNA template)	BamHI	XhoI
pGEX-6p-1/*SLD3 N0*	pGEX-6p-1	*FLAG-SLD3*	PCR (*S. cerevisiae* W303 genomic DNA template)	BamHI	XhoI
pGEX-6p-1/*SLD3 N4*	pGEX-6p-1	*FLAG-SLD3-1-435*	PCR (*S. cerevisiae* W303 genomic DNA template)	BamHI	XhoI
pGEX-6p-1/*SLD3 N5*	pGEX-6p-1	*FLAG-SLD3-1-585*	PCR (*S. cerevisiae* W303 genomic DNA template)	BamHI	XhoI
pGEX-6p-1/*SLD3 C0*	pGEX-6p-1	*SLD3-FLAG*	PCR (*S. cerevisiae* W303 genomic DNA template)	BamHI	XhoI

pGEX-6p-1/*SLD3 C1*	pGEX-6p-1	*SLD3-586-668-FLAG*	PCR (*S. cerevisiae* W303 genomic DNA template)	BamHI	XhoI
pGEX-6p-1/*SLD3 C2*	pGEX-6p-1	*SLD3-436-668-FLAG*	PCR (*S. cerevisiae* W303 genomic DNA template)	BamHI	XhoI
pGEX-6p-1/*SLD3 C3*	pGEX-6p-1	*SLD3-326-668-FLAG*	PCR (*S. cerevisiae* W303 genomic DNA template)	BamHI	XhoI
pGEX-6p-1/*SLD3 C4*	pGEX-6p-1	*SLD3-251-668-FLAG*	PCR (*S. cerevisiae* W303 genomic DNA template)	BamHI	XhoI
pGEX-6p-1/*SLD3 C5*	pGEX-6p-1	*SLD3-133-668-FLAG*	PCR (*S. cerevisiae* W303 genomic DNA template)	BamHI	XhoI
pGEX-6p-1/*SLD3 M3*	pGEX-6p-1	*SLD3-251-471-FLAG*	PCR (*S. cerevisiae* W303 genomic DNA template)	BamHI	XhoI
pGEX-6p-1/*SLD3 M4*	pGEX-6p-1	*SLD3-251-486-FLAG*	PCR (*S. cerevisiae* W303 genomic DNA template)	BamHI	XhoI
pGEX-6p-1/*SLD3 M5*	pGEX-6p-1	*SLD3-251-585-FLAG*	PCR (*S. cerevisiae* W303 genomic DNA template)	BamHI	XhoI
pGEX-6p-1/*SLD3 2E1*	pGEX-6p-1/*SLD3 N0*	*SLD3-2E1*	Synthetic construct (*SLD3-492-668)*	XbaI	XhoI
pGEX-6p-1/*SLD3 2E2*	pGEX-6p-1/*SLD3 N0*	*SLD3-2E2*	Synthetic construct (*SLD3-492-668)*	XbaI	XhoI
pGEX-6p-1/*SLD3 2E3*	pGEX-6p-1/*SLD3 N0*	*SLD3-2E3*	Synthetic construct (*SLD3-492-668)*	XbaI	XhoI
pGEX-6p-1/*SLD3 4E1*	pGEX-6p-1/*SLD3 N0*	*SLD3- 4E1*	Synthetic construct (*SLD3-492-668)*	XbaI	XhoI
pGEX-6p-1/*SLD3 4E2*	pGEX-6p-1/*SLD3 N0*	*SLD3- 4E2*	Synthetic construct (*SLD3-492-668)*	XbaI	XhoI
pGEX-6p-1/*SLD3 4E3*	pGEX-6p-1/*SLD3 N0*	*SLD3- 4E3*	Synthetic construct (*SLD3-492-668)*	XbaI	XhoI
pGEX-6p-1/*SLD3 6E*	pGEX-6p-1/*SLD3 N0*	*SLD3- 6E*	Synthetic construct (*SLD3-492-668)*	XbaI	XhoI
pGEX-6p-1/*SLD3 K301E*	pGEX-6p-1/*SLD3 C0*	*SLD3-K301E*	Synthetic construct (*SLD3-215-492)*	BsrG1	XbaI
pGEX-6p-1/*SLD3 3E1*	pGEX-6p-1/*SLD3 C0*	*SLD3- 3E1*	Synthetic construct (*SLD3-215-492)*	BsrG1	XbaI
pGEX-6p-1/*SLD3 3E2*	pGEX-6p-1/*SLD3 C0*	*SLD3- 3E2*	Synthetic construct (*SLD3-215-492)*	BsrG1	XbaI
pGEX-6p-1/*SLD3 4E4*	pGEX-6p-1/*SLD3 C0*	*SLD3- 4E4*	Synthetic construct (*SLD3-215-492)*	BsrG1	XbaI
pGEX-6p-1/*SLD3 4E5*	pGEX-6p-1/*SLD3 C0*	*SLD3- 4E5*	Synthetic construct (*SLD3-215-492)*	BsrG1	XbaI
pGEX-6p-1/*SLD3 8E*	pGEX-6p-1/*SLD3 C0*	*SLD3- 8E*	Synthetic construct (*SLD3-215-492)*	BsrG1	XbaI

pFA6a-natNT2/*SLD3 2E3*	pFA6a-natNT2	*SLD3-2E3*	PCR (pGEX-6p-1/*SLD3 2E3* template)	NdeI	PvuII
pFA6a-natNT2/*SLD3 6E*	pFA6a-natNT2	*SLD3- 6E*	PCR (pGEX-6p-1/*SLD3 6E* template)	NdeI	PvuII
pBP80-ADE2	pBP80	*ADE2*	PCR (*S. cerevisiae* W303 genomic DNA template)	PacI	SacI

* For details of cloning vectors not constructed in this study, see (Frigola et al., 2013, Janke et al., 2004, Longtine et al., 1998). pGEX-6p-1 was purchased from GE Healthcare. pBP80 is a modified version of pYM18 (Janke et al., 2004) containing a 3xFLAG tag associated with the *KanMX4* marker.

Appendix References

Frigola J, Remus D, Mehanna A, Diffley JFX (2013) ATPase-dependent quality control of DNA replication origin licensing. Nature 495: 339-43

Janke C, Magiera MM, Rathfelder N, Taxis C, Reber S, Maekawa H, Moreno-Borchart A, Doenges G, Schwob E, Schiebel E, Knop M (2004) A versatile toolbox for PCR-based tagging of yeast genes: new fluorescent proteins, more markers and promoter substitution cassettes. Yeast 21: 947-62

Longtine MS, McKenzie A, 3rd, Demarini DJ, Shah NG, Wach A, Brachat A, Philippsen P, Pringle JR (1998) Additional modules for versatile and economical PCR-based gene deletion and modification in Saccharomyces cerevisiae. Yeast 14: 953-61.

Randell JC, Fan A, Chan C, Francis LI, Heller RC, Galani K, Bell SP (2010) Mec1 Is One of Multiple Kinases that Prime the Mcm2-7 Helicase for Phosphorylation by Cdc7. Mol Cell 40: 353-63
