# Supplementary material for: Phosphopeptide binding by Sld3 links Dbf4‐dependent kinase to MCM replicative helicase activation
Source: EMBO J. 2016 Feb 24;35(9):961–73. doi: 10.15252/embj.201593552 (PMC4864760; doi:10.15252/embj.201593552)
Supplement: Supplementary file 2 — Expanded View Figures PDF [file EMBJ-35-961-s002.pdf]

Expanded View Figures

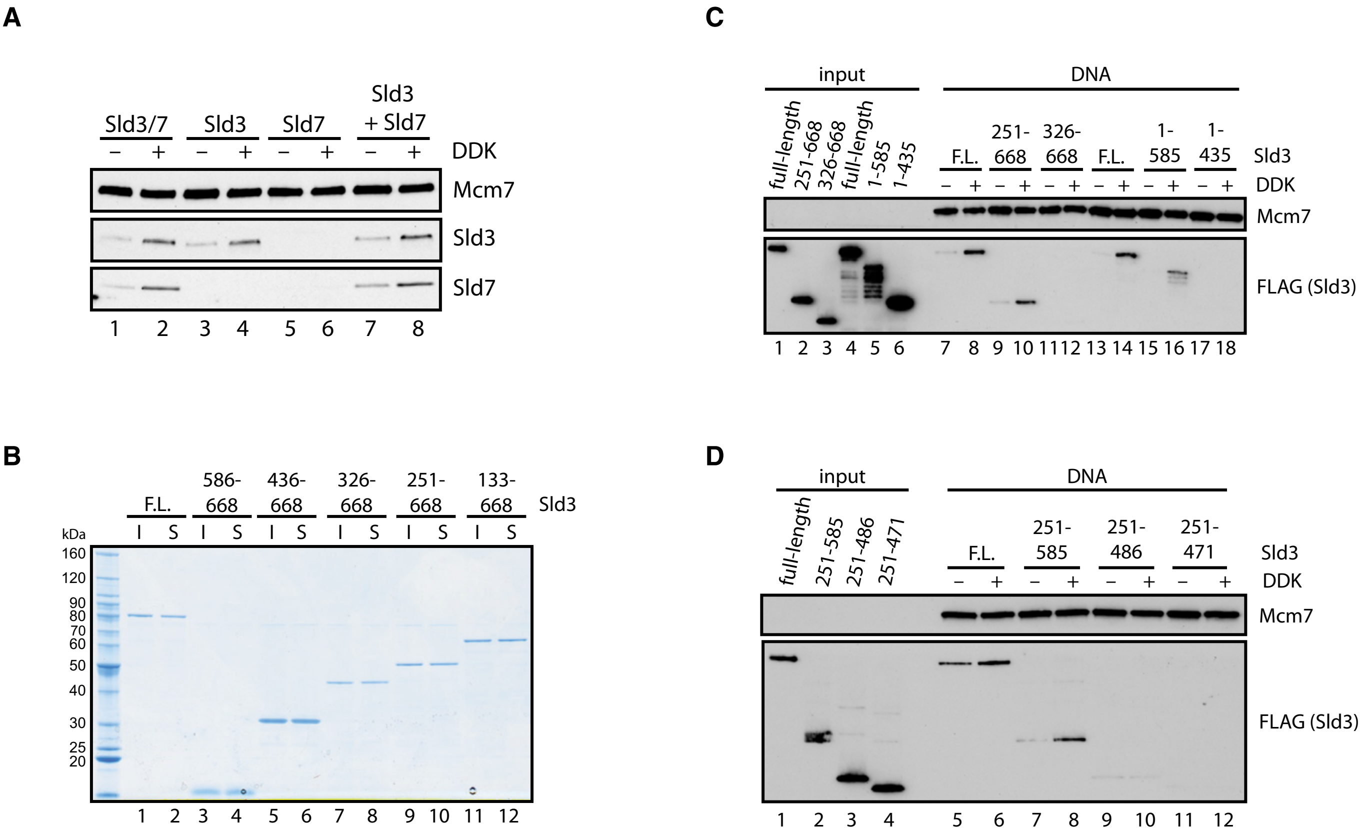

Figure EV1. Mapping the MCM-binding site in Sld3.

A Experiments performed as in Fig 2A, with Sld3/7 binding and wash steps performed at 0.5 M KOAc. Samples in lanes 1 and 2 included Sld3/7 purified from *S. cerevisiae*, whereas samples 3–8 used bacterially expressed Sld3 and Sld7.

B Full-length (F.L.) Sld3 and fragments visualised by SDS–PAGE and Coomassie staining. To test solubility, proteins were centrifuged at 21,000 *g* for 5 min. Input (I) and supernatant (S) samples taken before and after centrifugation, respectively.

C, D Sld3 recruitment assays performed as in Fig 2A using full-length (F.L.) or fragments of Sld3. These data are summarised in Fig 2C.

 © 2016 The Authors

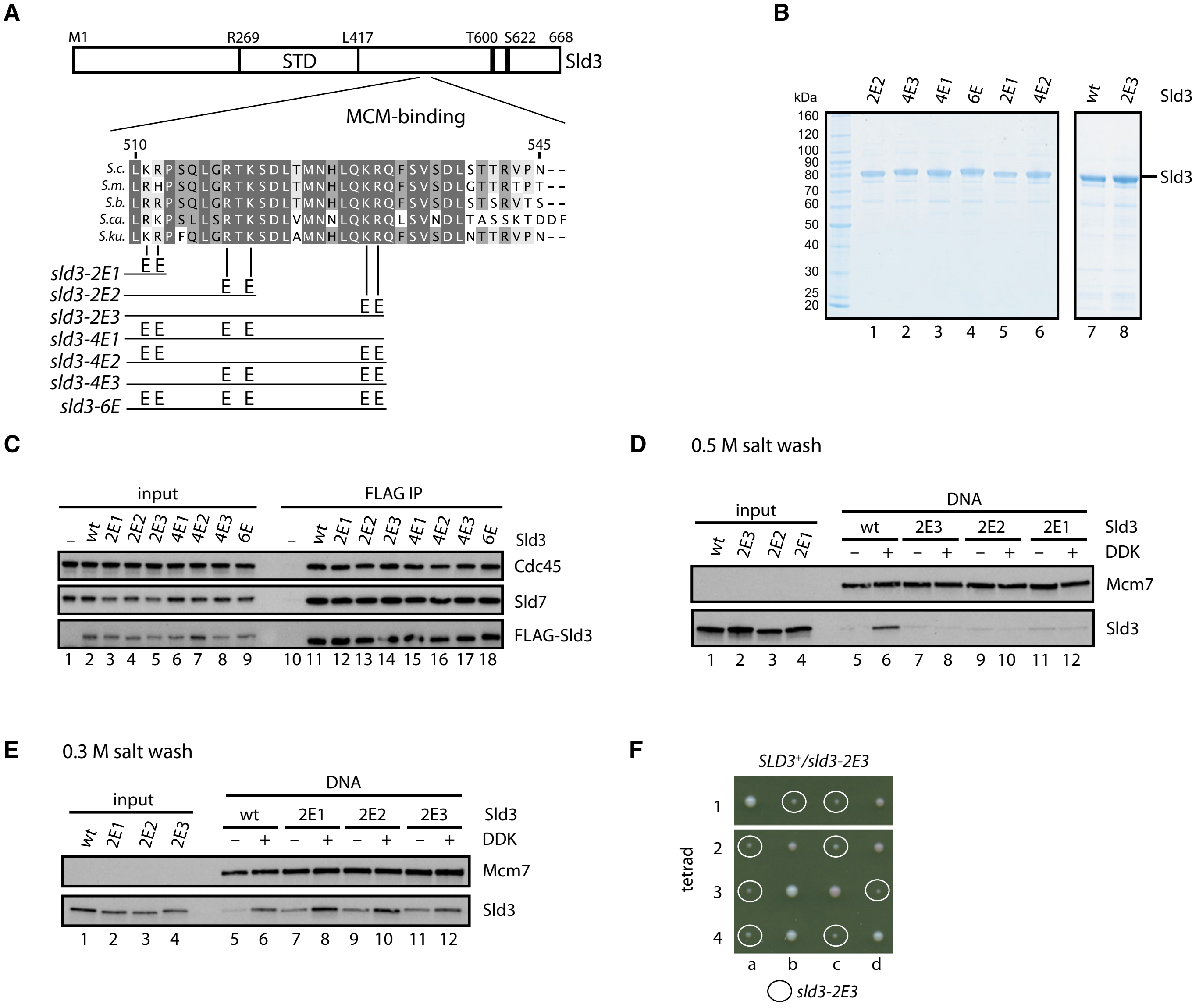

Figure EV2. Isolation of MCM-binding mutants in Sld3.

A Schematic showing the position of MCM-interacting residues in Sld3. Alignments were generated as in Fig 1E. Residues were substituted for Glu as indicated.

B Sld3 mutant and wild-type (wt) proteins visualised by SDS–PAGE and Coomassie staining.

C Wild-type or mutant FLAG-Sld3 was added to an S-phase protein extract and tested for interaction with Cdc45 and Sld7. Immunoprecipitated proteins were analysed by immunoblot.

D, E Sld3 recruitment experiments performed as in Fig 2A, with Sld3 binding and wash steps performed at 0.5 M KOAc (D) or 0.3 M K-glutamate (E).

F Representative tetrads from dissection of *SLD3*$^{+}$/*sld3-2E3* heterozygotes.

© 2016 The Authors

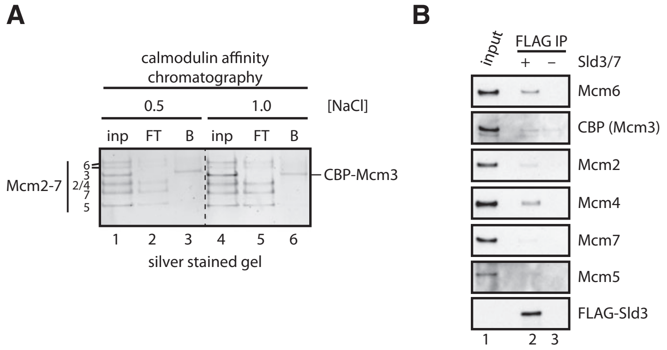

Figure EV3. Characterisation of Sld3/7 binding to Mcm4 and Mcm6.

A, B Reactions performed as described in Fig 4A. In (A), Mcm subunits that had dissociated from DNA were subjected to calmodulin affinity chromatography to isolate CBP-Mcm3 and associated proteins. Input (inp), flow-through (FT) and beads (B) samples were analysed by SDS–PAGE and silver staining.

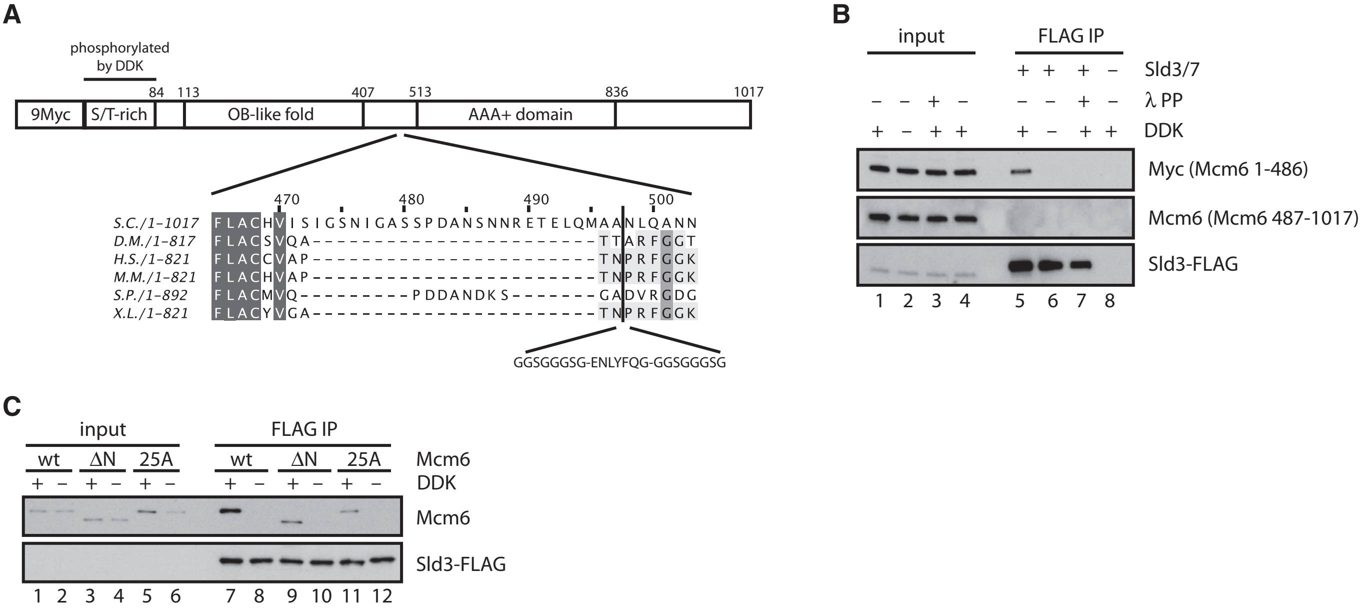

Figure EV4. Mapping the Sld3/7-interacting region in Mcm6.

A Schematic of TEV-cleavable Mcm6. The alignment was generated as in Fig 1E, including sequences from *Saccharomyces cerevisiae* (S.C.), *Drosophila melanogaster* (D.M.), *Homo sapiens* (H.S.), *Mus musculus* (M.M.), *Schizosaccharomyces pombe* (S.P.) and *Xenopus laevis* (X.L). Residue numbers correspond to S.C. Mcm6. Vertical line indicates the position where the TEV cleavage site and linker sequences were inserted.

B Reaction performed as described in Fig 4A using TEV-cleavable Mcm6. TEV protease was added concomitantly with buffer containing 0.5 M NaCl. The anti-Mcm6 antibody was raised against Mcm6 residues 997–1,017 (On *et al*, 2014).

C Reaction performed as in Fig 4A with wild-type (wt) or indicated mutant versions of Mcm6. Positions of mutations in Mcm6 are summarised in Fig EV5C.

Figure EV5. Identification of interactions between Sld3 and phosphorylated peptides of Mcm6 and Mcm4.

A Examples of Sld3-interacting peptides in Mcm6. 18-residue peptides (unmodified or phosphorylated at single Ser/Thr as indicated) covering Mcm6 residues 1–486 were spotted on membranes and incubated with FLAG-Sld3. Interactions were detected with an anti-FLAG antibody. Peptide sequences are shown to the right. Interacting Ser/Thr are shown in red.

B Table summarising Sld3-interacting phosphorylated residues in Mcm6. Column two indicates the local sequence surrounding the phosphorylated residue. Column three indicates the number of peptides containing a given phosphorylated residue that bound to Sld3. Interacting Ser/Thr are shown in red.

C Table summarising Sld3/7 binding by Mcm6 mutants. Reactions were performed as in Fig 4A. ++ reflects wild-type binding, + reflects a partial defect in binding, and – reflects the absence of any detectable interaction with Sld3/7.

D–F Reactions performed as in Fig 4A, using the indicated wild-type (wt) or mutant versions of Mcm6. DDK was included at 50 (+) or 250 (++) nM as indicated.

G Examples of Sld3-interacting peptides in Mcm4. Experiment performed as in (A).

H Table summarising Sld3-interacting phosphorylated residues in Mcm4, organised as in (B). Residues labelled with * are mutated in *mcm4-25A*.

I Experiment performed as in Fig 4E.

▶

 © 2016 The Authors

A

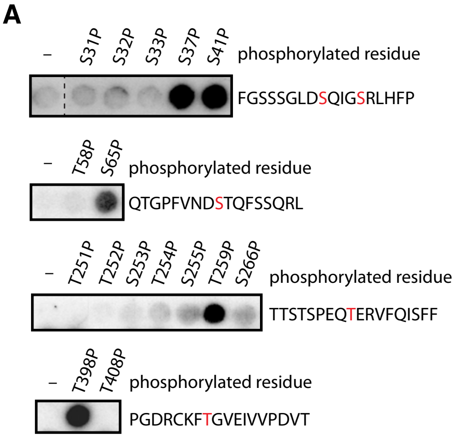

B

Site	Local sequence	Peptides
S37	SSGLDSQIGSR	4
S41	DSQIGSRLHFP	4
S65	PFVNDSTQFSS	3
T66	PFVNDSTQFSS	1
T150	IYDLNTIYIDY	3
S226	DDMNGSSLPRD	1
S227	DDMNGSSLPRD	1
T247	TSAMATRSITT	3
S255	ITTSTSPEQTE	1
T259	TSPEQTERVFQ	2
T272	FFNLPTVHRIR	1
S324	DNVEQSFKYTE	2
S351	LNVTRSRFLDW	1
T376	GSMPRTLDVIL	2
T398	DRCKFTGVEIV	2
T423	SSTLDTRGISK	1
S475	SPDANSNNRET	1
T480	SNNRETELQMA	2

C

Mutant	Mutations	Sld3/7 binding
WT	N/A	++
ΔN	Δ2-84	+
25A	S2A, S3A, T9A, S11A, S12A, S18A, S19A, S23A, S24A, S31A, S32A, S33A, S37A, S47A, S48A, S49A, S56A, S65A, T66A, S69A, S70A, T75A, S78A, S126A, T127A	+
12A	T150A, T247A, S255A, T259A, T272A, S324A, S351A, T376A, T398A, T423A, S475A, T480A	++
ΔN + 3A	Δ2-84, S255A, T259A, T272A	+
ΔN + Δ199-260	Δ2-84, Δ199-260	+
ΔN + 11A	Δ2-84, T247A, S255A, T259A, T272A, S324A, S351A, T376A, T398A, T423A, S475A, T480A	-

D

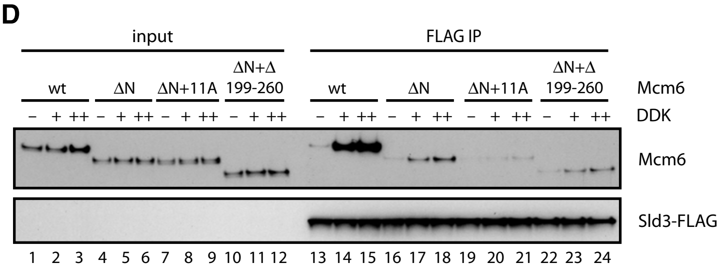

E

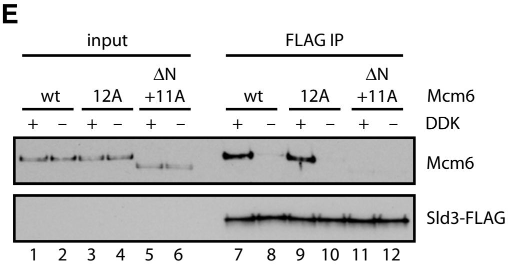

F

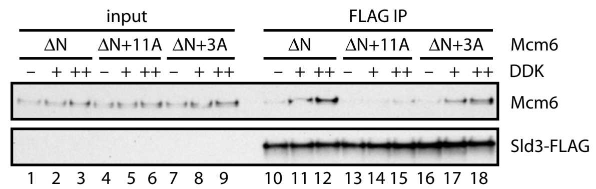

G

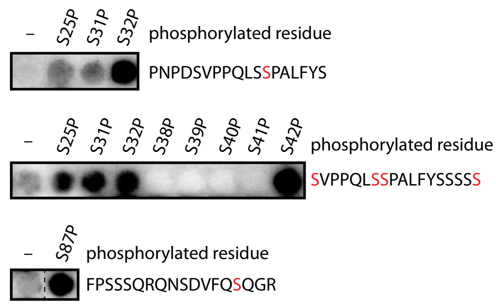

H

Site	Sequence	Peptides
S25	VPNPDSVPPQL	2
S31*	PPQLSSPALFY	3
S32*	PPQLSSPALFY	6
S38	PALFYSSSSSQ	1
S40	LFYSSSSSQGD	2
S42*	YSSSSSQGDIY	1
S76*	LNFPSSSQRQN	1
S77*	NFPSSSQRQNS	1
S87*	SDVFQSQGRQG	2
S119	RALPTSSSSLG	1
S120	ALPTSSSSLGR	1
S121	LPTSSSSLGRN	1

I

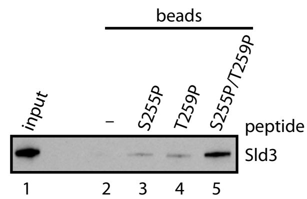

Figure EV5.

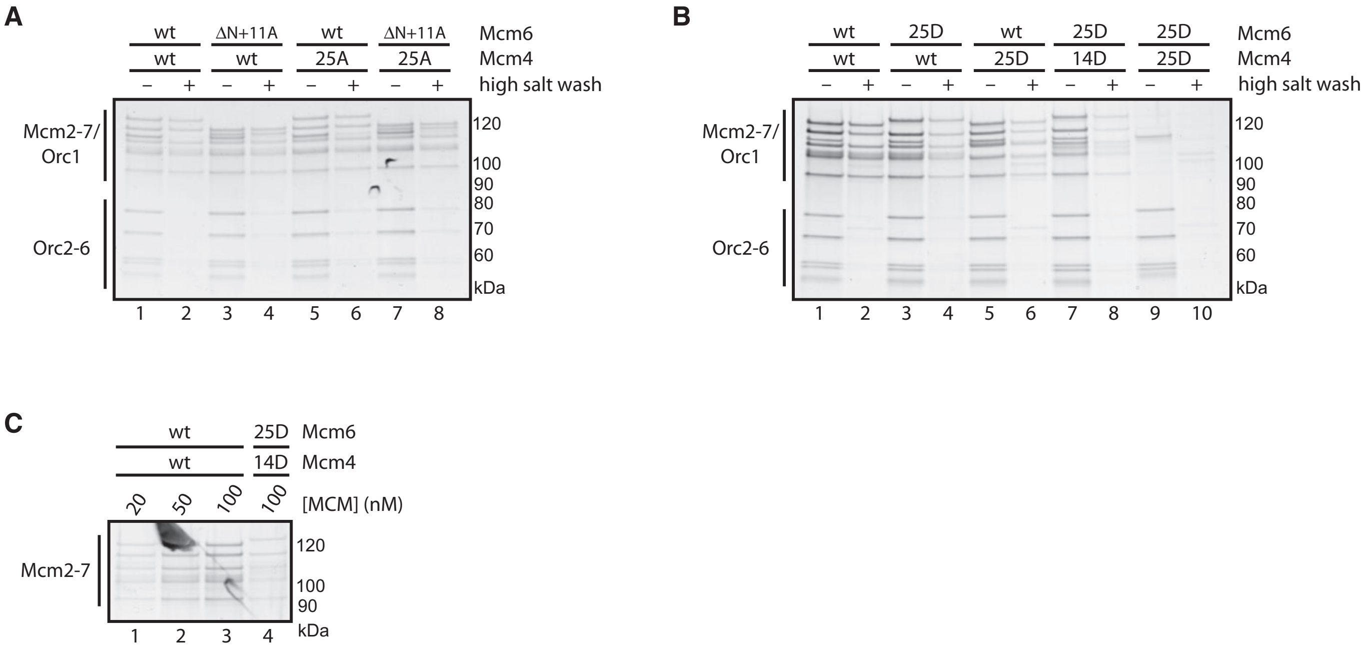

Figure EV6. MCM loading with Mcm4/Mcm6 mutants.

A–C MCM loading reactions using MCM containing the indicated wild-type, S/T-A (A) or S/T-D (B, C) versions of Mcm4 and Mcm6. DNA-bound proteins were eluted from beads by digestion of DNA with micrococcal nuclease and visualised by SDS–PAGE and silver staining.

 © 2016 The Authors

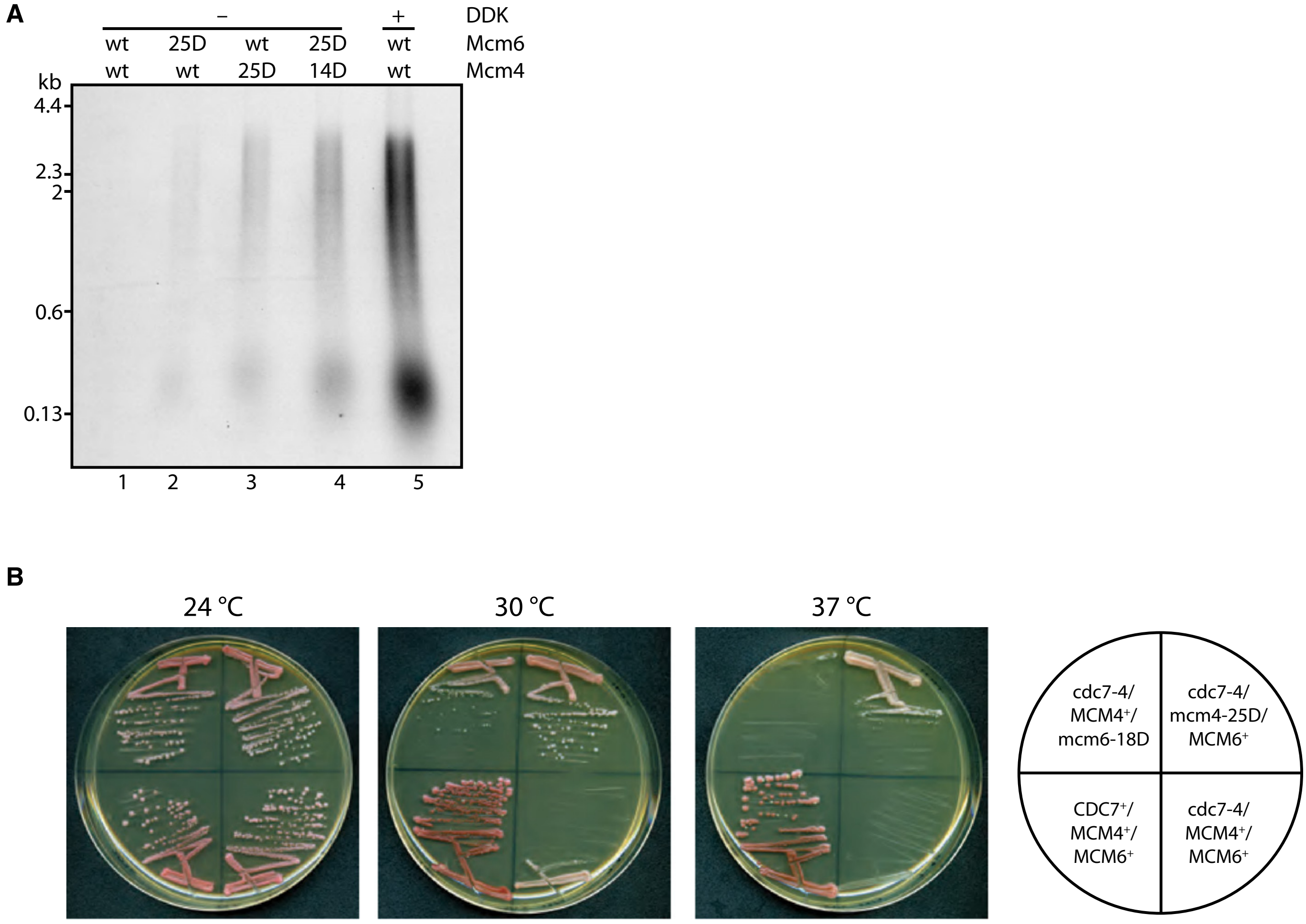

Figure EV7. DDK-independent replication with Mcm4/Mcm6 phosphomimicking mutants.

A Replication reaction performed as in Fig 1G using MCM containing wild-type (wt) or the indicated S/T-D substitution mutant versions of Mcm4 and Mcm6. MCM loading was not adjusted as it was for Fig 7D.

B Haploid yeast cells harbouring wild-type or mutant versions of *CDC7*, *MCM4* and *MCM6* were grown at the indicated temperatures. *MCM6-18D* was generated by targeting the endogenous *MCM6* locus with an *MCM6-25D* cassette derived from pTD75. We were unable to isolate a single transformant that carried all 25 mutations in *MCM6*, but could isolate a transformant (*MCM6-18D*) carrying a subset of these mutations.

© 2016 The Authors
